# Longitudinal academic achievement in children with a history of neonatal abstinence syndrome: a retrospective observational cohort study

**DOI:** 10.1016/j.lana.2026.101459

**Published:** 2026-03-25

**Authors:** Tammy E. Corr, Emily Wasserman, Eric W. Schaefer

**Affiliations:** a Penn State College of Medicine, Department of Pediatrics, Hershey, PA, USA; b Penn State College of Medicine, Department of Public Health Sciences, Hershey, PA, USA

**Keywords:** Neonatal abstinence syndrome, Neonatal opioid withdrawal syndrome, Substance exposed newborn, Opioid exposed newborn, Maternal substance use disorder, Academic achievement

## Abstract

**Background:**

Neonatal abstinence syndrome (NAS) has been associated with increased risks for adverse developmental, cognitive, and behavioral outcomes in childhood. However, there are limited data on academic achievement. We aimed to compare longitudinal academic achievement test scores in school-aged children with and without a history of NAS while controlling for relevant biologic and socioenvironmental variables.

**Methods:**

This retrospective, observational cohort study used a large, comprehensive, linked dataset from the South Carolina Integrated Data System to evaluate standardized English/Language Arts (ELA) and math achievement scores from grades 3–8 for children with and without a history of NAS. Linear mixed effects modeling assessed test scores by NAS group and grade, with random effects to account for correlation within school districts, schools, students, and mothers. Thirteen bio-socio-environmental variables were also included in the models.

**Findings:**

The analysis sample included 3494 students: 814 (23%) children with a history of NAS and 2680 (77%) without, frequency matched in a 1:3 ratio according to birth year, sex, mother's education level, and insurance payer at birth. 30.5% had a mother with less than a high school education and 85.1% were insured by Medicaid/uninsured. Mean test scores between students with and without a history of NAS were similar for ELA (−6.3 points, [95% confidence interval (CI): −15.1 to 2.5], *p* = 0.16) and math (−8.9 points, [95% CI: −16.9 to −0.9], *p* = 0.030).

**Interpretation:**

In a sample of children matched on bio-socio-environmental factors and for whom potential confounders were controlled, we found similar academic achievement scores between children with and without a history of NAS. These results emphasize the importance of socioenvironmental factors on childhood outcomes and suggest prenatal opioid exposure and NAS contribute minimally to academic achievement.

**Funding:**

This work was supported by funding from the National Institute on Drug Abuse 5K23DA055096 award to Dr. Tammy Corr.

**Research in context**

**Evidence before this study**

Neonatal abstinence syndrome (NAS) now affects 12.4 of every 1000 Medicaid births in the United States. While the existing literature indicates that children with a history of NAS and prenatal substance exposure may remain susceptible to developmental and academic challenges well into childhood, methodological shortcomings in accounting for confounding variables known to influence developmental and educational outcomes weaken the validity of the findings and conclusions drawn. A state-of-the art review article on the specific topic of prenatal opioid exposure and neurodevelopmental outcomes was published in September 2019 and highlighted the existence of conflicting results, an inability to draw conclusions on neurodevelopmental outcomes following prenatal opioid exposure, and the need for longitudinal study designs that accounted for confounding factors. Building upon these findings, the current authors searched PubMed and Ovid from July 2019–June 2024 for all relevant articles on child development and academic performance following prenatal opioid exposure and/or NAS/neonatal opioid withdrawal syndrome. Search terms included “neonatal abstinence syndrome” or “neonatal opioid withdrawal syndrome” and synonyms of “developmental outcomes” and “academic achievement”. There were 14 additional human studies on the associations of NAS and developmental outcomes identified during this time frame: 7 which suggested worse developmental or academic consequences and 7 that had mixed results or noted difficulty in drawing conclusions due to confounding factors. Therefore, it remains unclear whether NAS contributes to poor neurodevelopmental outcomes and academic achievement.

**Added value of this study**

This study utilized a large, integrated state data system to evaluate longitudinal academic achievement of school-aged children with a history of NAS in the context of maternal and child health, home socioeconomics, the home environment, and the school environment. The results demonstrate that children with a history of NAS perform similarly on longitudinal academic achievement tests to children without a history of NAS when matched on relevant biological and socioenvironmental factors, though, notably, all children in our matched cohorts, who were socioeconomically disadvantaged and often born to mothers with low education levels, performed worse than the state average. These findings challenge the existing evidence that implies prenatal opioid exposure and NAS significantly contribute to poor neurodevelopmental and academic outcomes among children with a history of NAS.

**Implications of all the available evidence**

Studies on the effects of prenatal opioid exposure and NAS on long-term neurodevelopment, including academic achievement, remain inconsistent. However, there is growing acknowledgement that longitudinal studies assessing developmental and academic success of children with a history of *in utero* opioid exposure must consider potential confounding variables. In studies that do consider these confounders, it is clear that socioenvironmental factors strongly contribute to developmental and academic outcomes and should be a standard consideration when evaluating sequalae of opioid exposure and NAS over evaluating either in isolation. Consequently, the findings of these studies also emphasize the essential role of social supports in optimizing future academic achievement.

## Introduction

Rates of neonatal abstinence syndrome (NAS), a withdrawal condition in newborns with prenatal opioid exposure with or without other psychotropic substances,^1^ remain significantly elevated in the U.S. and abroad, and NAS affected 12.4 of every 1000 Medicaid birth hospitalizations in 2020.^2^^,^^3^ While the immediate postnatal effects of prenatal opioid exposure have been established and include neonatal withdrawal,^4^ increased length of birth hospitalization,^5^ and associations with preterm delivery^6^ and lower birth weight,^6^^,^^7^ less is known about the long-term effects of *in utero* opioid exposure.

Studies suggest that neonates with prenatal opioid exposure are at increased risk for adverse cognitive, psychomotor, and behavioral outcomes as toddlers and preschoolers,^8^^,^^9^ and these negative associations last well into childhood.^10^ Children with a history of NAS may remain susceptible to ongoing challenges into adolescence with reports of increased special education use^11^ and deficient and deteriorating academic performance from early school years through high school.^12^ While these studies contribute to a growing body of literature showing an association between prenatal opioid exposure and adverse neurodevelopmental, behavioral, and educational outcomes in childhood, methodological shortcomings in controlling confounding variables known to influence developmental and educational outcomes weaken the validity of the findings and conclusions drawn.^13^^,^^14^

Furthermore, the term “NAS” has evolved over the years, and this evolution has contributed to the confusion in interpreting outcomes related to prenatal opioid exposure and neonatal opioid withdrawal. Coined in the 1970s, the term NAS initially referred to symptoms of neonatal withdrawal from prenatal exposure to heroin and, subsequently, methadone. Over the years, a diagnosis of “NAS” has grown to indicate neonatal withdrawal secondary to prenatal exposure to other illicit opioids such as fentanyl, *in utero* opioid exposure through medical therapy for pain conditions, opioid exposure via maternal medication for opioid use disorder (MOUD), and either isolated or co-exposure to other psychotropic medications.^15^ In addition, the term NAS is often used non-specifically by providers in practice and in medical records to indicate neonatal withdrawal, prenatal opioid exposure, or observation for suspected *in utero* substance exposure. These inconsistencies prompted the introduction and adoption of the term neonatal opioid withdrawal syndrome (NOWS) and prenatally opioid exposed (POE) newborn in the 2010's as well as efforts to standardize the definition of the term NAS, which still functions to indicate prenatal opioid exposure with or without additional psychotropic medications.^1^ Despite these recent clarifications, interpreting previous studies that evaluate the effects of NAS, and specifically prenatal opioid exposure with postnatal withdrawal symptoms, on child development remains difficult for researchers, clinicians, and families.

Mu opioid receptor agonists (i.e. buprenorphine, methadone) are the recommended treatment for maternal opioid use disorder (OUD) and can directly result in NAS/NOWS. Therefore, understanding the relationship between prenatal opioid exposure and academic performance is important to mitigate concerns over future potential risks to children and ensure appropriate, evidence-based care is adopted by mothers with OUD. Likewise, pregnant persons with OUD who engage in illicit opioid use and those utilizing opioids for chronic pain control are likely to be equally concerned about the association of chronic *in utero* opioid exposure and future scholastic achievement. Therefore, this study aims to assess the association between NAS/NOWS and longitudinal academic achievement in school-aged children after controlling for relevant biologic and socioenvironmental variables.

For the purposes of this study, the authors have chosen to use the broader term “NAS” to indicate neonatal opioid withdrawal due to prenatal exposure to opioids with or without additional psychotropic medications.

## Methods

### Study cohort

This study was a retrospective, observational cohort study. The cohort was drawn from a large, comprehensive state data warehouse, the South Carolina Integrated Data System (SCID),^16^ which links data from various SC state agencies, non-profits, and private entities. SCID identified all children with a history of NAS born between 2003 and 2014 who enrolled in SC public schools between 2017 and 2023 using International Classification of Diseases, 9th and 10th revision (ICD-9/10) codes 779.5 (ICD-9) and P96.1, P04.14, P04.4, P04.40, and P04.49 (ICD-10) given in the first 7 days after birth. The SCID conducted a frequency match for these children to children without a history of NAS according to four stratification factors obtained from birth certificate records: birth year, sex, mother's education level at birth, and insurance type at birth such that the resulting distributions reflected a 1:3 ratio of all those with a history of NAS to a sample of those without for each stratification factor. The data were then transferred from the SCID via secure electronic file transmission to our institution.

Substantial data cleaning and processing were then required to further refine the transferred study sample following receipt by first restricting the cohort to students that contributed at least one standardized test score in English/Language Arts (ELA) and/or math during grades 3–8 for the 2017–2023 school years. Scores were extracted from the SC College-and Career-Ready Assessment, a vertical scale score (VSS) system that allows for the longitudinal comparison of student performance across grade levels and school years. The data were reduced so that each school year for a student was reflected once, with each year corresponding to a single grade, allowing for instances of repeat grades across school years wherever present. Children born at a gestational age <34 weeks, identified using SC Vital Records data, were excluded from the analysis to remove the known influence of prematurity on academic achievement.^17^^,^^18^ These refinements resulted in a matching ratio of 1 case to 3.29 controls.

### Statistical analysis

Linear mixed effects models were used to assess ELA and math VSS as a function of grade. The model contained fixed effects for NAS group, grade, school year, and stratification factors used for frequency matching with the exception of the birth year (a stratification factor) as it was almost perfectly associated with grade and school year. As recommended for matched cohort studies, the variables used in frequency matching were included in the models to reduce bias.^19^

A total of 13 potential confounding variables were also included as fixed effects in the models. These variables included the following measures from SC Vital Records data: child's gestational age, child's birth weight, maternal age at child's birth, maternal race/ethnicity, degree of prenatal care during the pregnancy (Kotelchuck Index), indication of mother's tobacco use during pregnancy, and maternal receipt of Women Infants Children (WIC) supplemental nutrition program at time of birth (data availability beginning in 2004) as well as maternal criminal conviction, child foster care placement, child participation in preschool and/or prekindergarten, child participation in an Individualized Education Program (IEP), school rating, and school poverty index (refer to Supplemental Table S1 for more details on how these variables were defined for analysis). For some variables included in the models, a small percentage (<5%) were missing. As is appropriate in data with small amounts of missingness, we used single imputation to impute missing values at the median (for continuous variables) or the most common response (for categorical variables).

The primary parameter of interest was the mean difference in scores between students with and without a history of NAS, conditional on all other effects in the model. An interaction effect between NAS group and grade was examined to allow for different mean estimates over grade by NAS group, but the parameters were highly non-significant for both models, and the terms were dropped. Missing scores were excluded when fitting each model, but our methods are likelihood-based, and, therefore, valid under common missing data assumptions.

Four random effects were included in the model to account for specific sources of variation (school districts, schools within districts, mothers, and students). These random effects accounted for correlation of students within the same school district, students within the same schools (nested within school districts), students with the same mother, and within students for scores across grades. A random intercept effect was used for students, which induced a compound symmetric structure over grade. Other random effects structures were explored but the models would not converge, likely due to the complicated random effects structures.

### Sensitivity analyses

Three separate sensitivity analyses were conducted with respect to repeated grade data. First, repeated grades were excluded. Second, repeated grades were adjusted for in the model by including an indicator for a repeat grade. Third, for students with repeated grades, the entire series of grades was imputed under an assumption that a repeat grade did not occur, and they followed the typical, consecutive grade trajectory. The models were separately fit for each of these approaches to compare the parameter estimate for the NAS group effect.

Further, given the limitations of determining NAS status based solely on ICD diagnostic codes, an additional sensitivity analysis was performed to explore the robustness of the NAS group classifications. Four additional layers of data were considered for this exploration (see Supplemental Table S2): 1) additional ICD diagnostic codes in the infant birth record indicative of prenatal substance exposure, 2) ICD diagnostic and procedure codes in the maternal record indicative of opioid use during the pregnancy, 3) maternal Medicaid pharmaceutical claims for opioid medications dispensed during the pregnancy, and 4) methadone disbursement service code or Diagnostic and Statistical Manual of Mental Disorders, fourth edition, (DSM-IV) codes consistent with opioid use disorder during pregnancy in the maternal Department of Alcohol and Other Drug Abuse Services record. For this sensitivity analysis model, children classified as having NAS were excluded if no further supporting indications of NAS were found across these four additional layers, and children not classified as having NAS were excluded if further supporting indications of potential NAS were found.

Statistical analyses were run using SAS software Version 9.4 (SAS Institute Inc., Cary, NC, USA). All hypothesis tests were two-sided using a significance level of 0.05. As all subject data were deidentified, this study received an exemption status by Penn State's Institutional Review Board, STUDY00019884.

### Role of the funding source

The National Institute on Drug Abuse played no role in the study design, data collection, data analysis, interpretation of data, writing of this report, or in the decision to submit this manuscript for publication.

## Results

The final analysis sample included 3494 students, 814 (23%) of whom had a diagnostic history of NAS and 2680 (77%) of whom did not. All children with NAS were found to have the ICD-9 diagnostic code 779.5 as their NAS indicator, given the timing of births in our cohort (prior to 2015 when ICD-10 was widely implemented in the U.S.). Although the initial cohort spanned years of birth from 2003 to 2014, those born in the latter part of 2014 were effectively removed from the analysis set during data processing as those students were enrolled as third graders after the 2023 school year. The distributions across year of birth, sex, mother's education level, and health insurance payer were similar between the two groups due to the study design, and there were more cases of NAS noted over time, consistent with national trends of maternal opioid use disorders and NAS rates through 2017^2^^,^^20^ (Table 1). The sample was predominantly Medicaid insured/uninsured (85.1%; 2972 children), also congruent with national trends for prenatally opioid-exposed newborns, and 30.3% of the children (1060) were born to mothers with less than a high school degree (Table 1). Mothers of children with a history of NAS were predominantly non-Hispanic white (85.3%; 694) compared to a more balanced non-Hispanic distribution of white and black race among mothers of children without a history of NAS (Table 1). Inadequate prenatal care was common among mothers of children with a history of NAS (46.7%; 375 mothers) as was tobacco use during pregnancy (60.4%; 492 mothers) (Table 1). Children with a history of NAS were more likely to have a mother with a criminal conviction (14.5% [118 of 814 children] vs 1.6% [44 of 2680 children] without an NAS history) and to be placed in foster care (20.5% [167 of 814] vs 4.2% [113 of 2680]) (Table 1). 762 (22.4%) of all students in the study attended a school with an unsatisfactory or below average rating, and the median poverty index score (percent of school students who meet South Carolina's school definition for living in poverty) was 72.4 (Table 1).

**Table 1:** Matching and potential confounding variables.

	NAS^a^ (N = 814)	No NAS (N = 2680)	Total (N = 3494)
Matching variables			
Year of birth, n (%)			
2003	18 (2.2%)	67 (2.5%)	85 (2.4%)
2004	40 (4.9%)	127 (4.7%)	167 (4.8%)
2005	40 (4.9%)	124 (4.6%)	164 (4.7%)
2006	60 (7.4%)	194 (7.2%)	254 (7.3%)
2007	45 (5.5%)	156 (5.8%)	201 (5.8%)
2008	56 (6.9%)	182 (6.8%)	238 (6.8%)
2009	73 (9.0%)	223 (8.3%)	296 (8.5%)
2010	83 (10.2%)	270 (10.1%)	353 (10.1%)
2011	92 (11.3%)	299 (11.2%)	391 (11.2%)
2012	121 (14.9%)	395 (14.7%)	516 (14.8%)
2013	121 (14.9%)	382 (14.3%)	503 (14.4%)
2014	65 (8.0%)	261 (9.7%)	326 (9.3%)
Child sex, n (%)			
Male	445 (54.7%)	1495 (55.8%)	1940 (55.5%)
Female	369 (45.3%)	1185 (44.2%)	1554 (44.5%)
Maternal education, n (%)			
Less than high school	248 (30.5%)	812 (30.3%)	1060 (30.3%)
High school graduate or GED^b^	259 (31.8%)	860 (32.1%)	1119 (32.0%)
Some college	267 (32.8%)	862 (32.2%)	1129 (32.3%)
College degree or higher	39 (4.8%)	135 (5.0%)	174 (5.0%)
Unknown	1 (0.1%)	11 (0.4%)	12 (0.3%)
Payer, n (%)			
Medicaid/Self-pay	693 (85.1%)	2279 (85.0%)	2972 (85.1%)
Insurance/HMO^c^/Tricare	121 (14.9%)	401 (15.0%)	522 (14.9%)
Covariates			
Maternal WIC^f^ recipient, n (%)			
Yes	498 (62.6%)	1856 (71.1%)	2354 (69.1%)
No	283 (35.6%)	721 (27.6%)	1004 (29.5%)
Unknown	14 (1.8%)	35 (1.3%)	49 (1.4%)
Missing	19	68	87
Maternal age at delivery, years			
N	814	2680	3494
Mean (SD^d^)	27.7 (5.32)	25.0 (5.56)	25.6 (5.63)
Median	27.0	24.0	25.0
IQR^e^	24.0, 31.0	21.0, 28.0	21.0, 29.0
Range	15.0, 43.0	14.0, 46.0	14.0, 46.0
Maternal race/ethnicity, n (%)			
White, non-Hispanic	694 (85.3%)	1182 (44.1%)	1876 (53.7%)
Black, non-Hispanic	102 (12.5%)	1148 (42.8%)	1250 (35.8%)
White, Hispanic	13 (1.6%)	293 (10.9%)	306 (8.8%)
Other	5 (0.6%)	57 (2.1%)	62 (1.8%)
Adequacy of prenatal care^g^, n (%)			
Inadequate	375 (46.7%)	624 (23.4%)	999 (28.6%)
Intermediate	68 (8.5%)	179 (6.7%)	247 (7.1%)
Adequate	132 (16.4%)	677 (25.3%)	809 (23.2%)
Adequate plus	228 (28.4%)	1192 (44.6%)	1420 (40.9%)
Missing	11	8	19
Prenatal tobacco use, n (%)			
Yes	492 (60.4%)	417 (15.6%)	909 (26.0%)
No	322 (39.6%)	2261 (84.4%)	2583 (73.9%)
Unknown	0 (0.0%)	2 (0.1%)	2 (0.1%)
Gestational age, weeks			
Mean (SD)	38.0 (1.79)	38.6 (1.43)	38.5 (1.54)
Median	38.0	39.0	39.0
IQR	37.0, 39.0	38.0, 40.0	38.0, 39.0
Range	34.0, 43.0	34.0, 43.0	34.0, 43.0
Birth weight, grams			
Mean (SD)	2946.8 (531.06)	3223.6 (514.08)	3159.2 (531.06)
Median	2927.5	3232.0	3175.0
IQR	2580.0, 3317.0	2902.5, 3545.0	2807.0, 3515.0
Range	1344.0, 4785.0	1190.0, 5075.0	1190.0, 5075.0
Foster care placement, n (%)			
Yes	167 (20.5%)	113 (4.2%)	280 (8.0%)
No	647 (79.5%)	2567 (95.8%)	3214 (92.0%)
Maternal criminal conviction, n (%)			
Yes	118 (14.5%)	44 (1.6%)	162 (4.6%)
No	696 (85.5%)	2636 (98.4%)	3332 (95.4%)
School rating^h^, n (%)			
Unsatisfactory	42 (5.3%)	189 (7.3%)	231 (6.8%)
Below average	101 (12.8%)	430 (16.5%)	531 (15.6%)
Average	245 (30.9%)	971 (37.3%)	1216 (35.8%)
Good	200 (25.3%)	573 (22.0%)	773 (22.8%)
Excellent	204 (25.8%)	442 (17.0%)	646 (19.0%)
Missing	22	75	97
School poverty index^i^			
N	792	2606	3398
Mean (SD^c^)	66.7 (17.97)	70.1 (17.57)	69.3 (17.72)
Median	69.5	73.2	72.4
IQR^d^	56.3, 79.8	59.8, 84.1	58.5, 83.6
Range	9.3, 97.0	9.6, 97.6	9.3, 97.6
Preschool participation^j^, n (%)			
Yes	372 (48.8%)	1531 (59.6%)	1903 (57.1%)
No	390 (51.2%)	1039 (40.4%)	1429 (42.9%)
Missing	52	110	162
Individualized Education Program, n (%)			
Yes	288 (35.4%)	654 (24.4%)	942 (27.0%)
No	526 (64.6%)	2026 (75.6%)	2552 (73.0%)

a NAS = Neonatal Abstinence Syndrome.

b GED = Graduate Equivalency Degree; includes technical school.

c HMO = Health Maintenance Organization.

d SD = Standard deviation.

e IQR = Interquartile range.

f WIC = Women Infants Children supplemental nutrition program.

g Kotelchuck index.

h School rating results based on child's first year of data in SC Department of Education.

i Percent of school students meeting criteria for living in poverty; poverty index results based on child's first year of data in SC Department of Education.

j Preschool participation = preschool and/or pre-kindergarten.

The majority of students (2827; 80.9%) had standardized test scores for at least 2 school years and nearly two-thirds had scores for 3 or more years (Table 2). While 667 students (19.1%) had only 1 test score, 598 of these (90.0%) were in grade 3 in 2023 or grade 8 in 2017—an artifact of the retrospective data sample accompanying the study design. ELA and math VSS were missing for less than 5% of the sample, and approximately 1% of the scores reflected a repeated grade (Table 2).

**Table 2:** Test score distribution.

	NAS^a^ (N = 2272)	No NAS (N = 7596)	Total (N = 9868)
By test score			
School year, n (%)			
2017	219 (9.6%)	763 (10.0%)	982 (10.0%)
2018	275 (12.1%)	903 (11.9%)	1178 (11.9%)
2019	323 (14.2%)	1066 (14.0%)	1389 (14.1%)
2020^e^	–	–	–
2021	410 (18.0%)	1414 (18.6%)	1824 (18.5%)
2022	482 (21.2%)	1602 (21.1%)	2084 (21.1%)
2023	563 (24.8%)	1848 (24.3%)	2411 (24.4%)
Grade, n (%)			
3	546 (24.0%)	1795 (23.6%)	2341 (23.7%)
4	475 (20.9%)	1540 (20.3%)	2015 (20.4%)
5	396 (17.4%)	1401 (18.4%)	1797 (18.2%)
6	340 (15.0%)	1151 (15.2%)	1491 (15.1%)
7	285 (12.5%)	960 (12.6%)	1245 (12.6%)
8	230 (10.1%)	749 (9.9%)	979 (9.9%)
Missing ELA^b^ VSS,^c^ n (%)	101 (4.4%)	365 (4.8%)	466 (4.7%)
Missing math VSS, n (%)	103 (4.5%)	370 (4.9%)	473 (4.8%)
Repeated grade, n (%)	26 (1.1%)	62 (0.8%)	88 (0.9%)
**By student**	**(N = 814)**	**(N = 2680)**	**(N = 3494)**

Number of school years included, n (%)			
1	154 (18.9%)	513 (19.1%)	667 (19.1%)
2	176 (21.6%)	521 (19.4%)	697 (19.9%)
3	268 (32.9%)	889 (33.2%)	1157 (33.1%)
4	121 (14.9%)	421 (15.7%)	542 (15.5%)
5	92 (11.3%)	326 (12.2%)	418 (12.0%)
6	3 (0.4%)	10 (0.4%)	13 (0.4%)
Age at first test score			
Mean (SD^d^)	10.2 (1.3)	10.1 (1.3)	10.1 (1.3)

a NAS = Neonatal Abstinence Syndrome.

b ELA = English/Language Arts.

c VSS = Vertical Scale Scores.

d SD = Standard deviation.

e No testing done—year of COVID-19 pandemic shutdowns.

**Fig. 1:**
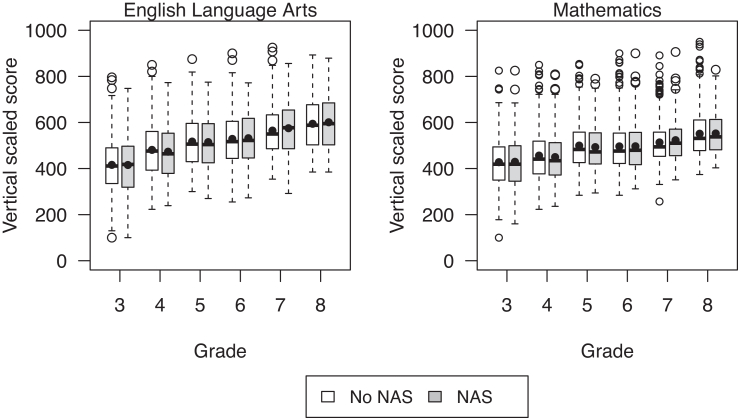
Box plots of scores by neonatal abstinence syndrome (NAS) and grade for English Language Arts and Mathematics vertical scale scores. Solid horizontal lines and circles inside the boxes reflect medians and means, respectively.

As expected for vertical scaled scoring, the average ELA and math standardized test scores generally increased across grades (Table 3, Fig. 1). Mean test scores of the sample were lower than the state mean scores for each grade (Table 3), while mean scores between the students with a history of NAS and controls were similar over time (Table 3, Fig. 1).

**Table 3:** Vertical scale scores by grade and neonatal abstinence syndrome diagnosis.

	Grade 3 (N = 2341)	Grade 4 (N = 2015)	Grade 5 (N = 1797)	Grade 6 (N = 1491)	Grade 7 (N = 1245)	Grade 8 (N = 979)
ELA^a^ VSS^b^						
NAS^c^, mean (SD)	416 (115)	473 (111)	515 (106)	532 (110)	575 (114)	601 (116)
No NAS, mean (SD)	416 (111)	481 (112)	517 (107)	529 (112)	565 (105)	594 (110)
State, mean (SD)	442 (117)	503 (120)	538 (111)	554 (117)	596 (115)	623 (116)
Math VSS						
NAS, mean (SD)	429 (113)	450 (100)	493 (97)	497 (102)	523 (87)	552 (92)
No NAS, mean (SD)	428 (105)	456 (103)	500 (99)	496 (102)	513 (87)	551 (97)
State, mean (SD)	456 (119)	484 (112)	529 (113)	527 (112)	546 (103)	581 (107)

a ELA = English/Language Arts.

b VSS = Vertical Scale Scores.

c NAS = Neonatal Abstinence Syndrome.

Based on the fitted linear mixed effects models, there was no significant association between NAS history and mean ELA scores (−6.3 points, [95% confidence interval (CI): −15.1 to 2.5]; *p* = 0.16) (Table 4). There was a small, albeit statistically significant, association between NAS history and math scores (−8.9 points, [95% CI: −16.9 to −0.9]; *p* = 0.030) (Table 4). The models demonstrated that scores for math and ELA increased with grade regardless of NAS history. Notably, scores also increased with increasing maternal education level and were significantly lower for children of non-Hispanic black mothers (Table 4). Maternal receipt of WIC supplemental nutrition, lower school rating, and participation in an IEP were all significantly associated with lower academic achievement test scores (Table 4). Medicaid insurance or uninsured status and inadequate prenatal care were associated with poorer performance in ELA testing only (Table 4).

**Table 4:** Parameter estimates from linear mixed effects models for English Language arts and math vertical scale scores.

Parameter	English Language Arts			Mathematics		
	Mean difference (SE^a^)	95% CI^b^	*p*-value	Mean difference (SE)	95% CI	*p*-value
Intercept	366.7 (46.9)	(274.8, 458.6)	<0.0001	431.7 (42.6)	(348.2, 515.1)	<0.0001
Grade						
3 (ref^c^)						
4	62.2 (1.8)	(58.7, 65.7)	<0.0001	28.5 (1.7)	(25.1, 31.9)	<0.0001
5	101.3 (2.0)	(97.3, 105.3)	<0.0001	73.5 (2.0)	(69.7, 77.4)	<0.0001
6	113.0 (2.5)	(108.1, 117.9)	<0.0001	70.4 (2.5)	(65.6, 75.2)	<0.0001
7	153.8 (2.8)	(148.3, 159.3)	<0.0001	89.4 (2.7)	(84.0, 94.7)	<0.0001
8	180.9 (3.2)	(174.5, 187.2)	<0.0001	124.1 (3.1)	(118.0, 130.2)	<0.0001
NAS^d^ group						
No NAS (ref)						
NAS	−6.3 (4.5)	(−15.1, 2.5)	0.16	−8.9 (4.1)	(−16.9, −0.9)	0.030
School year						
2017 (ref)						
2018	1.2 (2.3)	(−3.4, 5.8)	0.60	2.6 (2.3)	(−1.8, 7.1)	0.24
2019	3.1 (2.5)	(−1.8, 8.0)	0.21	0.6 (2.4)	(−4.2, 5.3)	0.81
2021	−8.0 (3.0)	(−13.9, −2.1)	0.0075	−22.6 (2.8)	(−28.2, −17.0)	<0.0001
2022	8.4 (3.2)	(2.1, 14.7)	0.0089	−10.8 (3.0)	(−16.8, −4.9)	0.0003
2023	33.6 (3.5)	(26.7, 40.4)	<0.0001	−7.5 (3.3)	(−13.9, −1.1)	0.021
Sex						
Male (ref)						
Female	24.5 (3.1)	(18.4, 30.5)	<0.0001	−3.2 (2.8)	(−8.7, 2.3)	0.24
Mother's race/ethnicity						
White, non-Hispanic (reference)						
Black, non-Hispanic	−49.1 (3.8)	(−56.5, −41.7)	<0.0001	−46.5 (3.5)	(−53.3, −39.7)	<0.0001
White, Hispanic	−4.5 (6.2)	(−16.7, 7.7)	0.47	−0.5 (5.7)	(−11.6, 10.6)	0.93
Other	33.6 (11.7)	(10.5, 56.6)	0.0043	44.1 (10.6)	(23.2, 64.9)	<0.0001
Maternal education						
Less than high school (ref)						
High school grad	12.5 (4.0)	(4.7, 20.4)	0.0017	14.2 (3.6)	(7.1, 21.3)	0.0001
Some college	36.1 (4.2)	(27.8, 44.3)	<0.0001	33.6 (3.8)	(26.1, 41.1)	<0.0001
Graduated from college	82.2 (8.2)	(66.1, 98.3)	<0.0001	79.3 (7.4)	(64.8, 93.9)	<0.0001
Unknown	−16.3 (25.9)	(−67.1, 34.5)	0.52	−2.6 (23.4)	(−48.5, 43.3)	0.91
Payer						
Insurance/HMO^e^/Tricare (ref)						
Medicaid/Self-pay	−10.7 (4.8)	(−20.0, −1.3)	0.025	−7.4 (4.3)	(−15.9, 1.0)	0.08
Maternal WIC^f^ recipient	−8.6 (3.7)	(−15.8, −1.4)	0.020	−10.7 (3.3)	(−17.2, −4.2)	0.0014
Maternal age, 1-year increase	0.01 (0.3)	(−0.6, 0.6)	0.99	−0.2 (0.3)	(−0.7, 0.4)	0.55
Adequacy of prenatal care^g^						
Inadequate	−16.0 (4.4)	(−24.6, −7.3)	0.0003	−6.7 (4.0)	(−14.5, 1.1)	0.09
Intermediate	−10.4 (6.6)	(−23.2, 2.5)	0.11	−3.5 (5.9)	(−15.2, 8.1)	0.55
Adequate (ref)						
Adequate plus	−10.9 (4.1)	(−18.8, −2.9)	0.0077	−7.0 (3.7)	(−14.2, 0.2)	0.06
Prenatal tobacco use	−7.4 (4.1)	(−15.5, 0.7)	0.07	−6.0 (3.7)	(−13.3, 1.3)	0.10
Gestational age, 1-week increase	1.9 (1.2)	(−0.4, 4.1)	0.10	1.3 (1.1)	(−0.7, 3.4)	0.20
Birth weight						
Low birth weight (<2500 g)	1.5 (5.8)	(−9.8, 12.8)	0.79	−0.6 (5.2)	(−10.8, 9.6)	0.90
Normal (ref)						
Macrosomia (>4000 g)	1.4 (7.2)	(−12.7, 15.4)	0.85	−2.1 (6.5)	(−14.9, 10.6)	0.74
Foster care placement	−0.8 (5.6)	(−11.8, 10.2)	0.88	−1.8 (5.1)	(−11.8, 8.3)	0.73
Maternal criminal conviction	−5.0 (6.7)	(−18.2, 8.1)	0.45	−1.3 (6.2)	(−13.5, 10.8)	0.82
Preschool participation^h^	−4.9 (3.2)	(−11.1, 1.3)	0.12	−1.8 (2.9)	(−7.5, 3.8)	0.52
Individualized Education Program (IEP)	−53.2 (3.1)	(−59.2, −47.1)	<0.0001	−42.1 (2.9)	(−47.8, −36.5)	<0.0001
School rating						
Unsatisfactory	−26.3 (4.1)	(−34.3, −18.3)	<0.0001	−37.4 (4.1)	(−45.4, −29.3)	<0.0001
Below average	−19.9 (3.1)	(−26.0, −13.8)	<0.0001	−28.2 (3.1)	(−34.3, −22.0)	<0.0001
Average	−11.0 (2.5)	(−16.0, −6.0)	<0.0001	−17.9 (2.5)	(−22.9, −12.9)	<0.0001
Good	−5.7 (2.4)	(−10.4, −1.0)	0.018	−9.6 (2.4)	(−14.3, −4.9)	0.0001
Excellent (ref)						
School poverty index, 10-point increase	−0.3 (0.7)	(−1.8, 1.2)	0.68	−0.5 (0.8)	(−2.0, 1.0)	0.52

Variance components for random effects		
Variance of mothers	3699.4	2893.6
Variance of school district	13.0	91.9
Variance of school	76.3	146.4
Variance of student	3081.6	2425.7

a SE = Standard error.

b CI = Confidence interval.

c Ref = Reference: group.

d NAS = Neonatal abstinence syndrome.

e HMO = Health Maintenance Organization.

f WIC = Women Infant Children supplemental nutrition program.

g Kotelchuck index.

h Preschool = preschool or pre-kindergarten programs.

The sensitivity analyses exploring the impact of repeated grades showed similar estimates for the NAS study group. For ELA scores, the mean differences (and standard errors) between NAS groups were −5.9 (4.5), −6.4 (4.5), and −6.3 (4.5) under each respective scenario (excluded, adjusted, imputed) compared to the estimates of −6.3 (4.5) reported in Table 4. Similarly, for math scores, the results were −8.6 (4.1), −9.0 (4.1), and −8.9 (4.1) for each respective scenario (excluded, adjusted, imputed) compared to estimates of −8.9 (4.1).

Finally, the sensitivity analysis evaluating academic achievement test scores in only those children for whom supporting indications were found to corroborate their assigned NAS classification (76.4% of original NAS group (n = 622); 85.5% of original non-NAS group (n = 2291)) demonstrated similar estimates for the two groups. Specifically, for ELA, the mean differences (and standard errors) between NAS groups were −3.8 (5.3) and for math −8.7 (4.8) compared to −6.3 (4.5) and −8.9 (4.1) reported for the original, primary model, supporting our earlier findings that there is no significant difference in ELA scholastic achievement test scores between children with and without a history of NAS and a statistically significant, but small decrease in math scholastic achievement test scores for children with a history of NAS.

## Discussion

Despite recent declines in NAS, rates of maternal opioid use remain elevated and rates of prenatal substance exposure continue to rise.^3^^,^^20^ The consequences of prenatal opioid exposure are poorly understood, and substantial efforts are underway to further this understanding.^21^ Our study found no significant difference in ELA and minor decreases in math achievement test scores among children in grades 3–8 based on their history of NAS after controlling for relevant biologic and socioenvironmental factors. These findings challenge the existing evidence that implies NAS intrinsically contributes to poor neurodevelopmental and academic outcomes.^9^, ^10^, ^11^, ^12^

While stigma around substance use disorders affects all individuals, pregnant persons with OUD are subjected to greater judgement and can harbor feelings of guilt.^22^ Even when actively receiving evidence-based treatment with medication for OUD, pregnant persons may be concerned over negative consequences of the medication itself, as both methadone and buprenorphine are known to cause NAS. Women who take opioids for chronic pain conditions face similar challenges.^23^ Concerns over these medication effects can be further compounded by existing studies that suggest potential long-term, adverse neurodevelopmental consequences for children with *in utero* opioid exposure.^22^ Therefore, the results of our investigation hold particular importance for families affected by addiction, those who require chronic opioids, and the providers who care for these populations. These findings should at least partially assuage parental fears over negative, long-term cognitive effects of NAS while encouraging pregnant persons with OUD to engage in appropriate treatment for their disease without added, unfounded guilt.

While there were minimal differences in academic achievement based on a diagnosis of NAS, the overall study sample—primarily Medicaid-insured/uninsured, with many children born to mothers with less than a high school degree—had scores below the state average across all grades. These observations are consistent with existing literature that demonstrates disadvantaged socioeconomic environments predict poorer neurodevelopmental outcomes and academic achievement, while more advantaged environments are associated with greater success.^24^ Indeed, the present analysis demonstrated significantly lower ELA and math academic achievement test scores in children who attended schools with lower school ratings and in children whose mothers received WIC supplementation, and significantly higher test scores with increasing degree of maternal education. Furthermore, children of non-Hispanic black mothers had significant and profoundly lower academic achievement test scores, highlighting the role of structural racism, systemic discrimination, and societal inequities in academic achievement. The cumulation of these results mirror previous work by Hurt and colleagues, who demonstrated that children with *in utero* cocaine exposure performed similarly to unexposed children with respect to cognitive testing longitudinally, but that children in her study, the majority of whom were from low-income families, had lower-than-average IQ's.^25^

Because school achievement is positively associated with adult productivity,^26^ it is important to address the underlying inequities that contribute to poor academic achievement in children to optimize potential. Economic disparities in income, race, parental education, and environmental resources directly impact student learning, childhood health, and school quality.^27^ Effective strategies will need to address existing prejudices and should provide community supports for young families, improved access to child and family healthcare, expanded entry into early childhood education, and equitable funding for schools regardless of geographic location or socioeconomic environment so that achievement gaps can be closed.^27^^,^^28^

For children with a history of NAS, specific legislative efforts and U.S. governmental agencies have aimed to address these needs through actions such as the 2016 amendment to the Children Abuse and Protection Act to include Plans of Safe Care for infants affected by prenatal substance exposure and publications such as the Substance Abuse and Mental Health Service Administration's “A Collaborative Approach to the Treatment of Pregnant Women with Opioid Use Disorders.” Central to these efforts is an emphasis on multidisciplinary care approaches that address health, social, and logistical needs for families.

Medical providers also play a key role in this process. Pediatricians are the first to identify developmental abnormalities and are often the first to diagnose or suspect behavioral or learning disorders. However, as previous work has demonstrated children with prenatal opioid exposure are less likely to attend recommended well-child visits,^29^ it is important to educate families on the importance of these visits as well as address any barriers to attending. It is also important that appropriate referrals are made for therapy and treatment, including engagement with Early Intervention programs and implementation of school special education services or independent learning plans to optimize long-term outcomes. Despite the demonstrated positive effects of these early interventions, less than one-half of eligible patients with a history of NAS enroll in Early Intervention programs,^30^ and only 10% of infants with NAS histories are referred to Early Intervention are ultimately evaluated for special education services in school.^11^ Our own results show that children with a history of NAS were less likely to have attended preschool programs and were more likely to require an IEP (Table 1). Therefore, clinicians should be cognizant of barriers to engagement with these services and proactively address foreseeable challenges.

While various interdisciplinary models have been implemented to improve the care of pregnant persons with OUD and their children, more research is necessary to identify risk and resilience factors that may mitigate adverse outcomes in children with a history of prenatal opioid exposure and NAS. Prospective, longitudinal cohort studies such as the Helping to End Addiction Long-term® (HEAL) HEALthy Brain and Child Development study aim to accomplish this goal through a multi-dimensional assessment of perinatal, postnatal, and childhood factors that may impact a child's health and development.^21^ The results of such a study, which is not exclusive to children with prenatal substance exposure, will build further on the findings contained within this report and will be important to the optimization of all children's potential development, future academic achievement, and ultimately, their adult well-being and productivity.

There are several limitations to this study. NAS classification was determined by using ICD diagnostic codes found in healthcare datasets. Administrative data for NAS have yielded inconsistent results in both accuracy and positive predictive value of ICD codes.^31^, ^32^, ^33^ However, the SCID's comprehensive, linked database allowed access to a variety of data sources that could be leveraged to corroborate the likelihood that the initial ICD-assigned diagnosis was accurate and representative of children with prenatal substance exposure and NAS. Though we planned to define NAS using both ICD-9 and 10 codes, because our cohort was limited to those born between 2003 and 2014, the use of ICD-10 codes was not yet routine in the U.S. and ICD-9 codes alone were identified. Therefore, it is unclear whether our results will stand with the newer codes, and this question will require additional study. However, given that we used additional modalities and robust explorations aside from NAS ICD codes to affirm prenatal substance exposure, we anticipate our results will persist. An additional limitation of a sample born as early as 2003 is that the care of infants affected by NAS, including medications used in treatment as well as the widespread implementation of Eat Sleep Console^34^ in recent years, has changed both the type and duration of postnatal opioid exposure. Simultaneously, the types of prenatal opioid exposures, both therapeutic and illicit, and the management of maternal opioid use disorder have shifted over time. These changes in maternal–fetal and neonatal exposures may limit the generalizability of our findings, particularly in the recent era of increased fentanyl exposure. Finally, our results do not evaluate the type(s) of *in utero* substance exposure, potentially increasing the risk of residual confounding. Understanding the type (illicit vs prescription, opioid-only vs polysubstance exposure), timing (first vs third trimester) and duration of prenatal exposure, along with their subsequent effects on outcomes, is important to untangle. Therefore, our next steps and future studies will investigate these questions. The findings of these investigations hold particular relevance in the setting of an ongoing opioid epidemic.

In conclusion, in a sample of children matched on bio-socio-environmental factors, we found similar academic achievement test scores between children with and without a history of NAS. These results emphasize the importance of socioenvironmental factors on childhood outcomes and suggest prenatal opioid exposure and NAS contribute minimally to academic achievement.

## Contributors

Dr. Tammy Corr conceptualized and designed the study, defined clinical data variables for analysis, interpreted results of analyses, drafted the initial manuscript, and critically reviewed and revised the manuscript.

Ms. Emily Wasserman organized, cleaned, and processed the data received, programmed data definitions, structured data for analyses, and critically reviewed and revised the manuscript.

Mr. Eric Schaefer carried out all data analyses and hypothesis tests and critically reviewed and revised the manuscript.

Ms. Wasserman and Mr. Schaefer have directly accessed and verified the underlying data reported in the manuscript.

All authors had full access to the data in the study and accept responsibility to submit for publication. All authors approved the final manuscript as submitted and agree to be accountable for all aspects of the work.

## Data sharing statement

Binding contracts with the South Carolina Revenue and Fiscal Affairs Office prohibit the investigators from data sharing publicly.

## Declaration of interests

The authors have no relevant conflicts of interest.
