# Supplementary material for: Longitudinal academic achievement in children with a history of neonatal abstinence syndrome: a retrospective observational cohort study
Source: Lancet Reg Health Am. 2026 Mar 25;58:101459. doi: 10.1016/j.lana.2026.101459 (PMC13050036; doi:10.1016/j.lana.2026.101459)
Supplement: Supplementary Tables [file mmc1.pdf]

Title: Longitudinal Academic Achievement in Children with a History of Neonatal Abstinence Syndrome: A Retrospective Observational Cohort Study

Supplemental Material Table of Contents

Supplemental Table 1. Bio-Socio-Environmental Covariate Definitions……………………………………………………page 2

Supplemental Table 2. Sensitivity Analysis Definitions………………………………………………………………..…….page 3

Supplemental Table 1. Bio-Socio-Environmental Covariate Definitions

Variable	Variable Source	Variable Description
Child enrolled/participated in preschool/prekindergarten program(s)	SC[1] Department of Education, First Steps	Binary indicator per student that delineated whether child participated in any preschool and/or prekindergarten program
Child met criteria for IEP[2]	SC Department of Education	Binary indicator that delineated whether student had a qualifying Education Finance Act[3] code denoting an Individualized Education Program for that school year
Mother criminal conviction	SC Department of Corrections	Binary indicator per school year that delineated whether the mother had a criminal conviction during that school year or any year prior dating back to pregnancy
Child foster care placement	SC Department of Social Services	Binary indicator per school year that delineated whether the child was placed in foster care during that school year or any year prior dating back to birth
School Rating[4]	SC School Report Cards	Yearly school performance ratings for elementary and middle schools measured on a 100-point scale categorized into five overall performance rating levels (unsatisfactory, below average, average, good, and excellent), with points earned across a variety of performance markers
School Poverty Index[4]	SC School Report Cards	Yearly index based on the percentage of students in the school who are homeless, in foster care, Medicaid-eligible, or qualified for SNAP[5] or TANF[6] in the prior years

[1]SC=South Carolina, [2]IEP=Individualized Education Plan, [3]Education finance codes are used by the SC Department of Education to classify pupils' qualifying special education diagnoses to determine funding needs for school districts; for more information on codes, please visit www.eoc.sc.gov/accountability-manuals, [4]For more information on school rating and poverty index, please see Accountability Manuels by school year at www.eoc.sc.gov/accountability-manuals, [5]SNAP=Supplemental Nutrition Assistance Program, [6]TANF=Temporary Assistance for Needy Families

Supplemental Table 2. Sensitivity Analysis Definitions

**Layer 1: Additional ICD[1] diagnostic codes in the infant birth record indicative of prenatal substance exposure**		
Diagnostic Codes Indicating Unspecified Prenatal Drug Exposure	ICD-9	760.7, 760.79
	ICD-10	P04.1, P04.18, P04.19, P04.8, P04.89, P04.9
Diagnostic Codes Indicating Prenatal Opioid Exposure	ICD-10	760.72
**Layer 2: ICD diagnostic and procedure codes in the maternal record during pregnancy indicative of substance use disorder**		
Diagnostic Codes Indicating Maternal Use of Drugs, Specific to Pregnancy	ICD-9	648.3, 648.30, 648.31, 648.32, 648.33, 648.34, 655.5, 655.50, 655.51, 655.53
	ICD-10	O35.5, O35.5XX0, O35.5XX1, O35.5XX2, O35.5XX3, O35.5XX4, O35.5XX5, O35.5XX9, O99.32, O99.320, O99.321, O99.322, O99.323, O99.324, O99.325
Diagnostic Codes Indicating Maternal Use of Drugs, Unspecified	ICD-9	292, 292.0, 292.1, 292.11, 292.12, 292.2, 292.8, 292.81, 292.82, 292.83, 292.84, 292.85, 292.89, 292.9, 304, 304.6, 304.60, 304.61, 304.62, 304.63, 304.8, 304.80, 304.81, 304.82, 304.83, 304.9, 304.90, 304.91, 304.92, 304.93, 305, 305.9, 305.90, 305.91, 305.92, 305.93, 946.00, E950, E950.4, E950.5, E950.9, E980, E980.4, E980.5, E980.9
	ICD-10	R78.4, R82.5, V65.42, X43, X44, X63, X64, X85, Y13, Y14, Z50.3, Z71.5, Z71.51, Z72.2, Z78.4
Diagnostic Codes Indicating Maternal Use of Opioids	ICD-9	304.0, 304.00, 304.01, 304.02, 304.03, 304.7, 304.70, 304.71, 304.72, 304.73, 305.5, 305.50, 305.51, 305.52, 305.53, 965, 965.0, 965.00, 965.01, 965.02, 965.09, 970.1, E850, E850.0, E850.1, E850.2, E935.0, E935.1, E935.2, E940.1
	ICD-10	F11, F11.1, F11.10, F11.11, F11.12, F11.120, F11.121, F11.122, F11.129, F11.13, F11.14, F11.15, F11.150, F11.151, F11.159, F11.18, F11.181, F11.182, F11.188, F11.19, F11.2, F11.20, F11.21, F11.22, F11.220, F11.221, F11.222, F11.229, F11.23, F11.24, F11.25, F11.250, F11.251, F11.259, F11.28, F11.281, F11.282, F11.288, F11.29, F11.9, F11.90, F11.91, F11.92, F11.920, F11.921, F11.922, F11.929, F11.93, F11.94, F11.95, F11.950, F11.951, F11.959, F11.98, F11.981, F11.982, F11.988, F11.99, R78.1, T40, T40.0, T40.0X, T40.0X1, T40.0X1A, T40.0X1D, T40.0X1S, T40.0X2, T40.0X2A, T40.0X2D, T40.0X2S, T40.0X3, T40.0X3A, T40.0X3D, T40.0X3S, T40.0X4, T40.0X4A, T40.0X4D, T40.0X4S, T40.0X5, T40.0X5A, T40.0X5D, T40.0X5S, T40.0X6, T40.0X6A, T40.0X6D, T40.0X6S, T40.1, T40.1X, T40.1X1, T40.1X1A, T40.1X1D, T40.1X1S, T40.1X2, T40.1X2A, T40.1X2D, T40.1X2S, T40.1X3, T40.1X3A, T40.1X3D, T40.1X3S, T40.1X4, T40.1X4A, T40.1X4D, T40.1X4S, T40.2, T40.2X, T40.2X1, T40.2X1A, T40.2X1D, T40.2X1S, T40.2X2, T40.2X2A, T40.2X2D, T40.2X2S, T40.2X3, T40.2X3A, T40.2X3D, T40.2X3S, T40.2X4, T40.2X4A, T40.2X4D, T40.2X4S, T40.2X5, T40.2X5A, T40.2X5D, T40.2X5S, T40.2X6, T40.2X6A, T40.2X6D, T40.2X6S, T40.3, T40.3X, T40.3X1, T40.3X1A, T40.3X1D, T40.3X1S, T40.3X2, T40.3X2A, T40.3X2D, T40.3X2S, T40.3X3, T40.3X3A, T40.3X3D, T40.3X3S, T40.3X4, T40.3X4A, T40.3X4D, T40.3X4S, T40.3X5, T40.3X5A, T40.3X5D, T40.3X5S, T40.3X6, T40.3X6A, T40.3X6D, T40.3X6S, T40.4, T40.4, T40.41, T40.411, T40.411A, T40.411D, T40.411S, T40.412, T40.412A, T40.412D, T40.412S, T40.413, T40.413A, T40.413D, T40.413S, T40.414, T40.414A, T40.414D, T40.414S, T40.415, T40.415A, T40.415D, T40.415S, T40.416, T40.416A, T40.416D, T40.416S, T40.42, T40.421, T40.421A, T40.421D, T40.421S, T40.422, T40.422A, T40.422D, T40.422S, T40.423, T40.423A, T40.423D, T40.423S, T40.424, T40.424A, T40.424D, T40.424S, T40.425, T40.425A, T40.425D, T40.425S, T40.426, T40.426A, T40.426D, T40.426S, T40.49, T40.491, T40.491A, T40.491D, T40.491S, T40.492, T40.492A, T40.492D, T40.492S, T40.493, T40.493A, T40.493D, T40.493S, T40.494, T40.494A, T40.494D, T40.494S, T40.495, T40.495A, T40.495D, T40.495S, T40.496, T40.496A, T40.496D, T40.496S, T40.4X, T40.4X1, T40.4X1A, T40.4X1D, T40.4X1S, T40.4X2, T40.4X2A, T40.4X2D, T40.4X2S, T40.4X3, T40.4X3A, T40.4X3D, T40.4X3S, T40.4X4, T40.4X4A, T40.4X4D, T40.4X4S, T40.4X5, T40.4X5A, T40.4X5D, T40.4X5S, T40.4X6, T40.4X6A, T40.4X6D, T40.4X6S, T40.6, T40.60, T40.601, T40.601A, T40.601D, T40.601S, T40.602, T40.602A, T40.602D, T40.602S, T40.603, T40.603A, T40.603D, T40.603S, T40.604, T40.604A, T40.604D, T40.604S, T40.605, T40.605A, T40.605D, T40.605S, T40.606, T40.606A, T40.606D, T40.606S, T40.69, T40.691, T40.691A, T40.691D, T40.691S, T40.692, T40.692A, T40.692D, T40.692S, T40.693, T40.693A, T40.693D, T40.693S, T40.694, T40.694A,

		T40.694D, T40.694S, T40.695, T40.695A, T40.695D, T40.695S, T40.696, T40.696A, T40.696D, T40.696S, T50.7, T50.7X, T50.7X1, T50.7X1A, T50.7X1D, T50.7X1S, T50.7X2, T50.7X2A, T50.7X2D, T50.7X2S, T50.7X3, T50.7X3A, T50.7X3D, T50.7X3S, T50.7X4, T50.7X4A, T50.7X4D, T50.7X4S, T50.7X5, T50.7X5A, T50.7X5D, T50.7X5S, T50.7X6, T50.7X6A, T50.7X6D, T50.7X6S, X42, X62, Y12
Procedure Codes Indicating Maternal Use of Drugs, Unspecified	ICD-9 PCS[2]	94.45, 94.54, 94.64, 94.65, 94.66, 94.67, 94.68, 94.69
	HCPCS[3]	G0396, G0397, H0001, H0003, H0005, H0006, H0007, H0008, H0009, H0010, H0011, H0012, H0013, H0014, H0015, H0016, H0022, H0049, H0050, H2034, H2035, H2036,
	ICD-10 PCS	HZ2ZZZZ, HZ30ZZZ, HZ31ZZZ, HZ32ZZZ, HZ33ZZZ, HZ34ZZZ, HZ35ZZZ, HZ36ZZZ, HZ37ZZZ, HZ38ZZZ, HZ39ZZZ, HZ3BZZZ, HZ3CZZZ, HZ40ZZZ, HZ41ZZZ, HZ42ZZZ, HZ43ZZZ, HZ44ZZZ, HZ45ZZZ, HZ46ZZZ, HZ47ZZZ, HZ48ZZZ, HZ49ZZZ, HZ4BZZZ, HZ4CZZZ, HZ50ZZZ, HZ51ZZZ, HZ52ZZZ, HZ53ZZZ, HZ54ZZZ, HZ55ZZZ, HZ56ZZZ, HZ57ZZZ, HZ58ZZZ, HZ59ZZZ, HZ5BZZZ, HZ5CZZZ, HZ5DZZZ, HZ63ZZZ, HZ88ZZZ, HZ89ZZZ, HZ98ZZZ, HZ99ZZZ, S9475, T1006, T1007, T1009, T1010, T1012
Procedure Codes Indicating Maternal Use of Opioids	HCPCS	G2076, G2077, G2078, G2079, G2080, H0020, J0570, J0571, J0572, J0573, J0574, J0575, J0592, J2310, J2315, M1032, M1033, M1034, M1035, M1036, S0109
	ICD-10 PCS	HZ81ZZZ, HZ82ZZZ, HZ84ZZZ, HZ85ZZZ, HZ86ZZZ, HZ91ZZZ, HZ92ZZZ, HZ94ZZZ, HZ95ZZZ, HZ96ZZZ
**Layer 3: Medicaid pharmaceutical claims in the maternal record for opioid medications dispensed during pregnancy**		
Pharmaceutical claims for medications commonly used for opioid use disorder.	Dispensed during pregnancy, including the month of birth	buprenorphine, buprenorphine-naloxone, methadone, naltrexone
Pharmaceutical claims for opioids commonly used for chronic pain <u>or</u> medication for opioid overdose.	Dispensed during pregnancy, excluding month of birth[4]	fentanyl patch, hydrocodone ER[5], hydromorphone ER, morphine sulfate SR[6]/CR[7]/ER, naloxone, oxycodone CR/ER, oxycontin ER/SR, oxymorphone ER, tapentadol ER
Pharmaceutical claims for opioids commonly used for acute pain.	Dispensed during pregnancy, excluding month of birth[4]	acetaminophen-caffeine-dihydrocodeine, acetaminophen-codeine, acetaminophen-tramadol, butalbital-aspirin-caffeine-codeine, codeine, hydrocodone-acetaminophen, hydrocodone-ibuprofen, hydromorphone, meperidine, morphine, oxycodone, oxycodone-acetaminophen, oxycodone-ibuprofen, oxymorphone, pentazocine, propoxyphene, tapentadol, tramadol, tramadol-acetaminophen
**Layer 4: Methadone disbursement service during the pregnancy or DSM-IV[8] code consistent with opioid use disorder during the pregnancy in the maternal DAODAS[9] record**		
Methadone disbursement during pregnancy		Service type code of "methadone disbursement"
DSM-IV Axis I codes indicating maternal use of drugs, unspecified		2920, 29289, 2929, 30480, 30490
DSM-IV Axis I codes indicating maternal use of opioids		30400, 30550

[1]ICD=International Classification of Diseases, [2]PCS= Procedure Coding System, [3]HCPCS= Healthcare Common Procedure Coding System, [4]pharmaceutical claims for these medications dispensed <u>solely</u> during the month of birth were excluded as an indicator of prenatal opioid exposure due to likelihood of their use in the treatment of postpartum, operative pain,[5]ER=extended release, [6]SR=sustained release, [7]CR=controlled release, [8]DSM-IV= Diagnostic and Statistical Manual of Mental Disorders, Fourth Edition, [9]DAODAS= Department of Alcohol and Other Drug Abuse Services
